# The Curious Question of Exercise-Induced Pulmonary Edema

**DOI:** 10.1155/2011/361931

**Published:** 2011-03-30

**Authors:** Melissa L. Bates, Emily T. Farrell, Marlowe W. Eldridge

**Affiliations:** ^1^Critical Care Division, Department of Pediatrics, The University of Wisconsin, H6/551 600 Highland Avenue, Madison, WI 53792, USA; ^2^Departments of Biomedical Engineering and Kinesiology, The University of Wisconsin, H6/551 600 Highland Avenue, Madison, WI 53792, USA

## Abstract

The question of whether pulmonary edema develops during exercise on land is controversial. Yet, the development of pulmonary edema during swimming and diving is well established. This paper addresses the current controversies that exist in the field of exercise-induced pulmonary edema on land and with water immersion. It also discusses the mechanisms by which pulmonary edema can develop during land exercise, swimming, and diving and the current gaps in knowledge that exist. Finally, this paper discusses how these fields can continue to advance and the areas where clinical knowledge is lacking.

## 1. The Challenge of Lung Fluid Handling during Exercise

During exercise, the transport of oxygen across the pulmonary membrane increases from ~4 mL/kg/min at rest to over 75 mL/kg/min in endurance athletes performing maximal exercise [1]. A couple of key anatomical and physiological features allow this almost twentyfold increase in the transport of oxygen from the alveolar region to the capillary network. The interface between the alveolus and capillary is exquisitely thin such that the diffusion distance between the alveolus and a red blood cell is only 1 *μ*m [2]. The surface area for gas exchange is immense (~1 × 10^4^ cm^2^), and the delicate, thin-walled vasculature has the capacity to be distended and recruited in order to accept a 5-fold increase in the cardiac output during exercise. During high intensity exercise, in the face of increased blood flow and elevated capillary pressures, the lung is faced with the challenge of keeping the respiratory membrane intact and the alveolar surface dry.

Like in other tissues, fluid flux across the lung vasculature is thought to be determined by the balance between the vascular hydrostatic and oncotic pressures relative to the interstitial space. The Starling equation illustrates this as

(1) $$J_{v}=K_{f}\left(\left[P_{c}-P_{i}\right]-\sigma \left[\pi_{c}-\pi_{i}\right]\right),$$

where *J*_*v*_ is the net fluid absorption or filtration from the vasculature, *K*_*f*_ is a filtration coefficient that describes the permeability of the capillary to fluid and is determined in part by the capillary surface area, *P*_*c*_ and *P*_*i*_ are the capillary and interstitial hydrostatic pressures, *π*_*c*_ and *π*_*i*_ are the capillary and interstitial oncotic pressures, and *σ* is a reflection coefficient that corrects the oncotic pressure for the permeability of the capillary to large proteins. Considering the variables in the Starling equation, exercise-induced pulmonary edema could occur by the following mechanisms:

- an increase in the capillary hydrostatic pressure,
- an increase in *K*_*f*_ as a result of capillary wall damage or increased capillary surface,
- an inability of the lymphatics to sufficiently clear water extruded from the vessels.

Although the lung is perfused at much lower pressures compared to the systemic vasculature (12 versus 120 mmHg, systolic at rest), the lung must still accommodate a more than doubling of the pulmonary artery driving pressure. This increase in pulmonary artery pressure is largely determined by the increased left atrial pressure that results from increased venous return to a limited atrial volume. Indeed, eighty percent of the increase in pulmonary artery pressure is explained by an increase in left atrial pressure [3]. Elevated pulmonary artery and left atrial pressures, coupled with a decreased intrathoracic pressure during inspiration, translate to increased capillary transmural pressures and the exudation of fluid from the capillary to the airspace. 

In the resting human, fluid transitions from the vascular space to the interstitium at a rate of 0.3 mL/kg/hr [4] and is cleared by the lymphatic system. Studies in exercising sheep demonstrate that lung lymphatic flow can increase 7–10-fold. This increase requires hyperpnea to occur and translates to 150–210 mL/hr of fluid clearance in a 70 kg human [5]. It was once thought that the simple negative pressure gradient between the lymphatics and the thoracic duct, caused by decreased thoracic pressure during the inspiratory phase of exercise hyperpnea, causes the increase in lymph flow. However, the real mechanism of increased lymph flow may be more elegant. Similar to the systemic veins, the lung lymphatics are valved. Much like skeletal muscle contraction returns venous blood to the heart through a series of valved vessels (termed the “skeletal muscle pump”), inspiratory and expiratory pressure oscillations may serve to pump blood through the pulmonary lymphatics [6–8]. Additionally, the lymphatics may have peristaltic properties. The collecting lymphatics have a smooth muscle layer that can have periodic contractions, elevating the internal pressure several mmHg [9]. They also have alpha and beta adrenergic receptors, and the application of catecholamines can increase the lymphatic pressure to 20–30 mmHg [10, 11]. Circulating catecholamines are increased with exercise [12] and may play a role in lymphatic function. However, their effect on lymphatic flow is not known.

## 2. Does Exercise-Induced Pulmonary Edema Exist?

The question of whether pulmonary edema develops during exercise is controversial. Systematic experimental attempts to document postexercise pulmonary edema yield conflicting data, but several clinical case reports exist in the literature. Clinically relevant edema has been reported in an elite cyclist participating in a transcontinental race [10], three runners participating in marathon and ultramarathon races [13, 14], and in a single individual after cross-country skiing in the cold [15]. In a single case report, an apparently healthy man developed pulmonary edema after exercise [16]. Interestingly, a clinical examination revealed mitral valve prolapsed, and the authors suggest that this structural abnormality likely contributed to the edema formation. There may be a role for unrecognized valve dysfunction in other cases. Indeed, in patients with left ventricular systolic dysfunction, mitral valve dysfunction is associated with increased pulmonary vascular pressure, capillary barrier disruption, and exercise-induced pulmonary edema [17]. To date, there have been no comprehensive studies quantifying the annual incidence of postexercise pulmonary edema in individuals with and without previously identified cardiac abnormalities.

The impetus to suspect a high prevalence of subclinical interstitial edema postexercise comes primarily from data obtained using the multiple inert gas elimination technique (MIGET). The MIGET quantifies the degree to which ventilation and perfusion in the lung are well matched (V/Q) [18, 19]. V/Q mismatch increases with moderate to heavy exercise and persists even after exercise termination and the return to baseline cardiopulmonary function [20, 21]. Hopkins has proposed that the “most plausible” explanation for this is the formation of interstitial pulmonary edema, causing peribronchial and perivascular cuffing. 

Several of the case reports mentioned in this paper point to exercise-induced pulmonary edema as a cause of exercise-induced arterial hypoxemia, which occurs in at least 50% of healthy individuals exercising at sea level [22]. In these reports, where clinical signs of alveolar flooding were noted, hypoxemia is probably the result of edema. However, in most exercising individuals, the issue is less clear. V/Q mismatch, the major piece of evidence for exercise-induced pulmonary edema, is probably a minimal contributor to gas exchange deficits. The proportion of the alveolar-arterial oxygen difference explained by V/Q mismatch does not change from rest to heavy exercise [23], leaving diffusion limitation and right-to-left shunt as the remaining possible culprits [23, 24]. Indeed, the increase in V/Q mismatch does not impact gas exchange because the increase in ventilation exceeds that of perfusion, resulting in few very low V/Q compartments. Hopkins suggests that the reason gas exchange abnormalities are not predicted by V/Q mismatch is because interstitial and peribronchial edema does not progress to the point of alveolar edema and flooding in most individuals [25].

The limitation of translating V/Q measurements from the realm of explanation to causation is that the V/Q abnormalities measured by MIGET have never been related to direct measures of pulmonary edema in the same individuals postexercise. A recent study used a rapid infusion of saline (20 mL/kg) to increase interstitial lung water without elevating cardiac output. Although an increase in lung water was verified by impedence cardiography, there was no evidence of V/Q mismatch [26]. Importantly, there was no evidence of change in the index of ventilation distribution (LogSDV and the mean of V) despite changes in spirometric measurements consistent with the development of interstitial edema. In a study of subjects with a history of high altitude pulmonary edema (HAPE), V/Q mismatch with exercise at 3,800 m was no different than controls with no history of HAPE. Additional resting V/Q inequality has only been observed in participants of Operation Everest II at simulated altitudes ≥20,000 ft [27]. Finally, performing repeated bouts of exercise, an activity that would be expected to successively compound interstitial edema, does not increase V/Q mismatch [28].

Several studies have relied on the use of direct imaging modalities (MRI, CT, etc.) and thoracic impedance to demonstrate pulmonary edema postexercise with mixed results. Some studies using CT and MRI have found evidence of increased lung water after sustained, heavy exercise [29, 30], but these findings have not been consistently replicated. There are several potential pitfalls to using imaging and thoracic impedance to measure exercise-induced edema. Both can be confounded by the increase in total thoracic water caused by the increased blood volume in the lung immediately postexercise. Magnetic resonance and CT imaging require a change in posture which alters the distribution of blood flow and may confound the ability to visualize edema. Additionally, MRI is time consuming; during the time required to complete the imaging, low level edema may resolve. Computed tomography can be impractical to use in research studies because of risks associated with X-ray radiation exposure [31]. Finally, some have argued that direct imaging is not sensitive enough to visualize interstitial edema; in order to visualize edema, it must have progressed to the point of alveolar flooding [25]. Certainly, overt alveolar flooding with exercise is rare. The major challenge to demonstrating subclinical pulmonary edema lies in the lack of a reliable, sensitive, gold standard measurement.

The best evidence for exercise-induced pulmonary edema comes from studies looking for capillary stress failure in postexercise bronchalveolar lavage fluid (BALF). Pulmonary capillary stress failure and hemorrhage are estimated to occur at capillary transmural pressures ≥40 mmHg [32]. Capillary stress failure and frank alveolar bleeding have been well documented in exercising thoroughbred race horses, which can achieve estimated capillary pressures ≥100 mmHg [33, 34]. Eldridge et al. found evidence of capillary disruption in athletes after three short bouts of intense sea level and high altitude exercise by examining the BALF for the presence of red blood cells [35]. Red cells were found in all athletes' BALF postexercise, but not in the BALF of resting controls. Although the BALF of each athlete was positive for red cells, the number of red cells found (5.4 × 10^4^/mL) was orders of magnitude lower than those found in humans with a clinical diagnosis of high altitude pulmonary edema (2.6 × 10^6^/mL) or exercising horses (30–60 × 10^6^/mL) [36, 37]. Still, these data provide evidence that the respiratory membrane is disrupted with intense exercise in healthy, active adults. 

The lesson learned from many of these studies is that the normal lung appears well designed for sea-level exercise. In most individuals, water exudation into the interstitium is balanced by an increase in lymph flow, preventing the development of clinical pulmonary edema marked by dyspnea, end expiratory crackles, and the production of bloody, frothy sputum. Despite the case reports that exist in the literature, the development of clinically important pulmonary edema with exercise remains a rare event. It has been suggested that the rare individuals who do develop exercise-induced pulmonary edema had an unknown underlying pathology that made them more likely to develop pulmonary edema with exertion [38].

## 3. What Can We Learn from Immersion Pulmonary Edema?

Exercise in water places a unique set of stresses on the respiratory system. Immersion pulmonary edema with breath-hold diving, SCUBA diving, and cold water exercise has been well documented in the literature [39–41]. In a survey of 460 active SCUBA divers, 1.1% had a history consistent with the development of immersion pulmonary edema [42]. A survey of triathletes from the group USA Triathlon revealed that 1.4% of the members had symptoms suggestive of pulmonary edema after completing an event that contained a swimming component [43] In 2009, the Professional Association of Diving Instructors reported that 900,000 new diving certifications are issued by its members each year [44]. If 1% of the population experiences immersion pulmonary edema, then 900,000 new divers per year translates to 9,000 new individuals at risk for immersion pulmonary edema.

Different populations seem to be at risk for the development of immersion edema, depending on the type of immersion activity. Cases in athletes swimming at the surface tend to occur in young, fit individuals performing intense exercise. The maneuvers performed by elite breath-hold and military divers make them susceptible to pulmonary barotrauma and alveolar hemorrhage. Some have speculated that SCUBA divers, some of whom tend to be older, may have undiagnosed left ventricular dysfunction that would contribute to the development of edema [45]. However, SCUBA divers are subjected to many of the same stressors as breath-hold divers and surface swimmers and their edema may not depend entirely upon pre-existing cardiovascular pathology. The cause of immersion pulmonary edema is probably multifactorial. 

Immersion in upright, seated research participants redirects ~0.7 L of blood to the thorax and increases pulmonary artery systolic pressure [46]. This is augmented by immersion in cold water, which redirects blood flow to the thorax in order to preserve body temperature, and caused by vasoconstriction in the trunk and extremities [47]. Exercise further increases thoracic blood volume and left atrial, pulmonary artery, and capillary pressures, although there is substantial variability in pulmonary artery pressure with immersion exercise. Peacher et al. found a large degree of intersubject variability in pulmonary artery pressure with surface immersion exercise (16.0–39.6 mmHg) [48], although the rise in pulmonary artery pressure was higher than what is typically observed during land exercise [49]. These investigators speculated that this high variability may explain why some individuals are more susceptible to edema formation than others when performing the same activity. For example, in a case report of 30 Israeli soldiers performing a 2.4 km open water time trial, 8/30 developed overt edema marked by frothy sputum, dyspnea, and hemoptysis [50]. There is debate as to whether pulmonary artery pressure continues to rise during exercise as conflicting studies have shown it to both increase and decrease with increasing exercise duration [47, 48]. 

The addition of diving further increases blood redirection to the thorax in order to combat the effect of the increased pressure on lung volume. As predicted by Boyle's Law, the barometric pressure rises during descent, while the lung volume shrinks. Blood is redirected from the periphery to the thorax [51]. This increases the vascular hydrostatic pressure and counteracts the increased airway pressures. Ferrigno and Lundgren predict that the effects of “lung squeeze” during a breath-hold dive would be sufficient to elevate the capillary hydrostatic pressure 11–32 mmHg [52]. These pressures, certainly beyond the 40 mmHg needed to disrupt capillary integrity, are sufficient to cause edema given long enough exposure. 

Ventilation may also contribute to the formation of edema, although knowledge in this area is limited. There are no direct studies of the importance of ventilation in edema formation, and most of what is known is inferred from other areas. For example, hyperventilation is required to increase fluid clearance by the lymphatics [5]. With high intensity exercise, minute ventilation tends to be lower during prone swimming compared to treadmill running, and the ability to increase minute ventilation is limited by the need to coordinate breathing with swimming stroke [53]. This may limit the lymphatics' ability to clear fluid. Submersion to the neck causes a 3-fold decline in the expiratory reserve volume and, as a result, low lung volume breathing [54]. Hydrostatic compression of the thorax increases inspiratory resistance [55] and decreases maximum expiratory flow rates at lung volumes <60% of total lung capacity [56]. The addition of a regulator during SCUBA diving can further increase the respiratory effort needed to overcome the regulator's resistance by >20 cm H_2_O [57]. These limitations demand an increase in the work of breathing, and the enhanced negative intrathoracic pressure during inhalation supports fluid filtration and edema formation.

Finally, glottal closure or SCUBA regulator failure may contribute to edema formation. In a study of seven men submersed supine in only 1 ft of water while breathing through a rigid metal tube, three of the men terminated the study within the first 30 seconds of the initial submersion [54] with the complaint that the breathing tube was obstructed. It was later discovered that they were experiencing involuntary glottal closure, which could be overcome by not attempting to inhale during the first 15 seconds of submersion. Similar to the development of flash pulmonary edema in patients with laryngospasm or upper airway obstruction [58, 59], breathing against an involuntarily closed glottis or a malfunctioning SCUBA regulator may generate large negative intrathoracic pressures and disrupt the integrity of the respiratory membrane.

## 4. What Gaps Still Exist in Our Knowledge?

Collaboration with investigators studying immersion pulmonary edema may provide new abilities to answer the question of whether subclinical pulmonary edema forms in athletes ubiquitously with intense, land exercise. The major obstacle limiting our ability to more definitively answer the question using current approaches is the lack of a sufficiently sensitive method of detection. Certainly, if edema forms in the most exercising individuals on land, it is subclinical. Until a more sensitive detection method is developed, evidence for and against the formation of edema with land exercise will continue to be met with skepticism. 

It is surprising that the rapid infusion of saline does not produce V/Q inequality, considering that spirometric measurements of midexpiratory flow are altered in a manner consistent with interstitial edema [26]. It is possible that the edema affects the lung uniformly, but it is also possible that the MIGET measurement is not able to detect pulmonary edema with sufficient sensitivity. Nonetheless, we know that edema develops in a substantial fraction of divers performing water exercise. Several recent studies have been conducted in which invasive pulmonary hemodynamic measurements were made in human research participants performing hyperbaric water exercise [47, 48], offering insight into the mechanism of edema formation during submersion. Pulmonary edema has been observed in the laboratory in humans performing immersion exercise and has been verified by observing blood in the conducting airways below the vocal cords [39]. To determine whether MIGET is able to quantify V/Q inequality in patients with pulmonary edema, it would be valuable to make measurements in patients with varying degrees of active edema, which could be generated in the laboratory with immersion exercise. MIGET measurements have been made in immersion [60], but not in individuals with immersion pulmonary edema. 

While immersion pulmonary edema is currently a very exciting and active field of research, investigators studying immersion pulmonary edema have inferred that elevations in pulmonary artery and left atrial pressure must be the major cause of immersion pulmonary edema. Yet, they have not directly linked hemodynamic measures to quantified edema formation. It is important that future mechanistic studies include a quantitative measurement of edema formation to truly define the importance of any mechanism. Because immersion pulmonary edema is more overt, it may be possible to quantify it using magnetic resonance imaging. The development of comprehensive mathematical models using data from research participants with immersion pulmonary edema may be able to be extrapolated to predict the edema formation that would occur with land exercise.

Another opportunity to advance both fields may lie in a return to the use of animal models. The majority of measurements using MIGET have been made in exercising human research participants. However, Hopkins et al. previously established the Yucatan miniswine as a valid model for the investigation of V/Q mismatch with exercise [61]. Like humans, the miniswine develops reproducible V/Q inequality with exercise. A simple, but useful, question to ask would be if the V/Q inequality in the miniswine is associated with an increase in lung water by simple assessment of the lung's wet to dry weight in exercising and nonexercising animals. V/Q measurements could also be related to interstitial thickness and other morphometric measurements with and without exercise. Since pigs have been described as developing perivascular edema with intense exercise, it is conceivable that there might be a relation between MIGET measurements and histological findings [62] in the miniswine.

Studies of immersion edema in humans have recently focused on hemodynamic measurements, although there is little data regarding changes in the other Starling forces. Hyperbaric hyperoxia without immersion or exercise induces lung function changes consistent with edema formation [63]. How, for example, do the gas mixtures breathed by divers affect fluid filtration? We know little about the effect of immersion exercise on the reflection and filtration coefficients and less about the degree to which the lymphatics may be overwhelmed by fluid filtration during immersion exercise. Studies conducted using an exercising sheep model reveal the importance of the lymphatics in keeping the airspaces dry on land, although we still know very little about how they are regulated on land and in water. We know that adrenergic stimulation has the ability to increase pressure within the lymphatics by 30 mmHg, but we have little insight into how adrenergic mediators affect fluid clearance by the lymphatics. We also do not know how the changes in pulmonary mechanics and ventilation with immersion affect lymphatic function. Isolated lung and intact, large animals could be used to investigate the importance of the filtration and reflection coefficients and the regulation of the lymphatics.

## 5. Why Is It Important Clinically to Study Exercise-Induced Pulmonary Edema?

Each of these smaller gaps in our knowledge contributes to a larger gap; we have no real comprehensive mathematical models of fluid handling during exercise or understanding of the risk factors for the development of exercise-induced pulmonary edema. For example, we know that sex, small lung volume, low basal nitric oxide production, genetic susceptibility (specifically polymorphisms in the angiotensin converting enzyme gene), and a decreased hypoxic ventilatory response predispose individuals to exercise-induced pulmonary edema at altitude [35, 64–69], but we do not know the risk factors associated with land or immersion exercise. Mathematical models are valuable both to understand how fluid balance is maintained in the healthy lung, and also to predict fluid shifts in patients with cardiopulmonary pathology. Some have speculated that the rare patients who develop pulmonary edema with exercise on land must have some previously undetected left heart dysfunction [38]. Considering the association between heart failure and pulmonary edema, surely these patients would have warranted examination by echocardiography. How subtle must that dysfunction be to result in edema with exercise and why was it not detected when these patients presented with pulmonary edema? 

Water exercise is widely recommended for the elderly and patients with heart failure and systemic hypertension because of its low impact nature and the ability to tailor aquatic exercise to accommodate different fitness levels [70–72]. Yet we know nothing of the effect of immersion and exercise on lung fluid balance in these populations. Could low intensity water exercise contribute to edema formation in these groups? More studies are needed to begin to answer these questions.
